# Suppression of Midgut Microbiota Impact Pyrethroid Susceptibility in *Aedes aegypti*

**DOI:** 10.3389/fmicb.2022.761459

**Published:** 2022-08-01

**Authors:** Mayra A. Gómez-Govea, María de Lourdes Ramírez-Ahuja, Yamili Contreras-Perera, Armando J. Jiménez-Camacho, Gabriel Ruiz-Ayma, Olga Karina Villanueva-Segura, Gerardo de Jesús Trujillo-Rodríguez, Iván Delgado-Enciso, Margarita L. Martínez-Fierro, Pablo Manrique-Saide, Henry Puerta-Guardo, Adriana E. Flores-Suárez, Gustavo Ponce-García, Iram P. Rodríguez-Sánchez

**Affiliations:** ^1^Universidad Autónoma de Nuevo León, Facultad de Ciencias Biológicas, Laboratorio de Fisiología Molecular y Estructural, San Nicolás de los Garza, Mexico; ^2^Unidad Colaborativa de Bioensayos Entomológicos (UCBE) y del Laboratorio de Control Biológico (LCB) para Ae. aegypti, Universidad Autónoma de Yucatán (UADY), Mérida, Mexico; ^3^Universidad Autónoma de Nuevo León, Facultad de Ciencias Biologicas, Laboratorio de Biológía de la Conservación, San Nicolás de los Garza, Mexico; ^4^Universidad de Colima, Facultad de Medicina, Colima, Mexico; ^5^Universidad Autónoma de Zacatecas, Laboratorio de Medicina Molecular, Unidad Académica de Medicina Humana, Zacatecas, Mexico; ^6^Universidad Autónoma de Nuevo León, Facultad de Ciencias Biológicas, Departamento de Zoología de Invertebrados, San Nicolás de los Garza, Mexico

**Keywords:** microbiome, *Aedes aegypti*, insecticide susceptibility, deltamethrin, permethrin

## Abstract

Aedes *aegypti* is a mosquito that transmits viral diseases such as dengue, chikungunya, Zika, and yellow fever. The insect’s microbiota is recognized for regulating several biological processes, including digestion, metabolism, egg production, development, and immune response. However, the role of the bacteria involved in insecticide susceptibility has not been established. Therefore, the objective of this study was to characterize the resident microbiota in a field population of *A. aegypti* to evaluate its role associated with susceptibility to the insecticides permethrin and deltamethrin. Mosquitoes were fed 10% sucrose mixed with antibiotics and then exposed to insecticides using a diagnostic dose. DNA was extracted, and sequencing of bacterial 16S rRNA was carried out on Illumina^®^ MiSeq™. Proteobacteria (92.4%) and Bacteroidetes (7.6%) were the phyla, which are most abundant in mosquitoes fed with sucrose 10%. After exposure to permethrin, the most abundant bacterial species were *Pantoea agglomerans* (38.4%) and *Pseudomonas azotoformans-fluorescens-synxantha* (14.2%). *Elizabethkingia meningoseptica* (38.4%) and *Ps. azotoformans-fluorescens-synxantha* (26.1%) were the most abundant after exposure to deltamethrin. Our results showed a decrease in mosquitoes’ survival when exposed to permethrin, while no difference in survival when exposed to deltamethrin when the microbiota was modified. We found that the change in microbiota modifies the response of mosquitoes to permethrin. These results are essential for a better understanding of mosquito physiology in response to insecticides.

## Introduction

According to the World Health Organization (WHO), vector diseases represent 17% of the estimated emerging infectious diseases in the world (WHO, 2020). This results from vector’s geographical expansion, world transport, unplanned urbanization, and climate change (Jupatanakul et al., 2014). Viruses like dengue, zika, and chikungunya are the most important arboviruses, which are the leading cause of the emerging infectious diseases worldwide (Patterson et al., 2016). The main vectors of these diseases are mosquitoes belonging to the genus *Aedes* (Diptera: Culicidae) (Mayer et al., 2017). In Mexico, the control of these mosquitoes depends on integrated management with insecticides (CENAPRECE, 2018); however, as in many other countries, the effectiveness of vector control programs is compromised by the development of resistance to insecticides (Musso and Gubler, 2015). Moreover, there is concern about the environmental impact of this approach (Nkya et al., 2013; Smith et al., 2016). Recent studies have shown that insecticide resistance is a diverse and global process related to metabolic mechanisms (increased enzymatic activity) and resistance to the target site (Smith et al., 2016; Dada et al., 2019). These problems have created the need to develop alternative methods for controlling mosquito populations (Ramirez et al., 2012).

Previous research indicates that mosquitoes can host different bacterial communities that vary depending on sex, stage of development, and environmental factors (Cirimotich et al., 2010; Ramirez et al., 2012; Terenius et al., 2012; Coon et al., 2016). The functions of these microorganisms in the insects are correlated with nutrition, immune response, protection against pathogens, digestion, and development, among others (Gaio et al., 2011; Douglas, 2014). Multiple studies have addressed the bacterial community in *A. aegypti*, where species of the genera *Bacillus, Elizabethkingia, Enterococcus, Klebsiella, Pantoea, Serratia*, and *Sphingomonas* have been found (Terenius et al., 2012). Other studies have demonstrated the role of microbiota as a modulator of vector competence in *Anopheles* during infection with *Plasmodium* (Abdul-Ghani et al., 2012; Dennison et al., 2014). In *A. atropalpus*, the intestinal microbiota has been found to be associated with variations in survival, size, and egg production (Coon et al., 2016). In addition, new control strategies and environmentally friendly measures have focused on endosymbiotic bacteria such as *Wolbachia* to reduce or block the transmission of pathogens such as dengue, chikungunya, and Zika viruses (Bian et al., 2010; Bennett et al., 2019; Scolari et al., 2019). Recently, the microbiota of insects has been found to be involved in the detoxification processes (Aislabie and Lloyd-Jones, 1995; Itoh et al., 2018). Studies have shown that bacteria such as *Bacillus cereus, Enterobacter asburiae*, and *Pantoea agglomerans* can degrade acephate, an organophosphate in the diamondback moth *Plutella xylostella* (Kikuchi et al., 2012; van den Bosch and Welte, 2017). Knowing that the detoxification mechanism is a complex process that is regulated by metabolic (increase in enzyme activity) or genetic (mutations) systems, our study aimed to characterize the resident microbiota in *A. aegypti* mosquitoes and their potential role in their susceptibility to the insecticides: permethrin and deltamethrin.

## Materials and Methods

### Biological Material

The *A. aegypti* mosquitoes were obtained locally from San Lorenzo, which is located in the municipality of Umán in the state of Yucatán, Mexico. Larvae were reared under controlled and aseptic conditions in the rearing room: 28 ± 1°C, 80 ± 5% RH, and a 12:12 h light: dark photoperiod. Larvae were fed with a mixture of tilapia feed meal and yeast (90:10 ratio, respectively). The adult colonies were incubated at 27 ± 2°C, 75 ± 5% RH, and under a 12:12 h light: dark regime in the insectarium of the Collaborative Unit for Entomologic Bioassays (UCBE) at the Universidad Autónoma de Yucatán (UADY).

### Microbiota Suppression

Once the larval development cycle was completed, the pupae were transferred to beakers for the emergence of adults in rearing cages 25 cm × 25 cm × 25 cm (Bug Dorm-1) previously disinfected with 70% ethanol to maintain the aseptic conditions. At 24 h post-emergence, groups of 100–125 females were selected for each treatment. In the first treatment, the female mosquitoes were fed with sterilized 10% sucrose solution (denoted as group A). For the modification of the microbiome, female mosquitoes (100-125) were placed and fed with sterilized 10% sucrose solution with 10 U/ml for penicillin/streptomycin (Gibco Life Technologies) (denoted as group H: treated with penicillin/streptomycin). In other treatments, female mosquitoes (100-125) were placed and fed with sterilized 10% sucrose solution with 15 μg/ml gentamicin (Gibco Life Technologies) (denoted as I: treated with gentamicin). The antibiotic concentrations used in this study were previously reported by Dong et al. (2009). The treatments were done for 3 consecutive days and no mortality of mosquitoes was recorded during the assay. Microbiota of mosquitoes treated with antibiotics was analyzed to ensure changes in the microbiota.

### Mosquito Susceptibility to Pyrethroid Insecticides

After modification to antibiotics, groups of mosquitoes were used for susceptibility bioassays. These assays were performed according to the CDC bottle method bioassay (Brogdon and McAllister, 1998), with 20 to 25 females with microbiota modification and without modification. Survival against each insecticide was evaluated in six groups of mosquitoes as follows: B: exposed to permethrin (control group), C: treated with penicillin/streptomycin permethrin exposed, D: treated with gentamicin permethrin exposed, E: exposed to deltamethrin (control group), F: treated with penicillin/streptomycin deltamethrin exposed and G: treated with gentamicin deltamethrin exposed). Groups were exposed to the diagnostic dose of permethrin of 15 μg/bottle and deltamethrin of 10 μg/bottle (>95% purity; Chemservice, West Chester, PA, United States). Each bioassay consisted of four replicates per treatment and one control bottle without insecticide. The bioassays were carried out as described above (average temperature of 27 ± 2°C and relative humidity of 75 ± 5%, under aseptic conditions). The specimens that survived the diagnostic time (TD, 30 min for both pyrethroids) were transferred to recovery cups. The gut was then extracted according to the method described by Liu et al. (2018) with modifications. Briefly, before dissection, adult insects were sterilized with 96% ethanol for 3 min, then rinsed 3 times with sterile deionized water. The guts of 20–25 adult females were dissected on a plate containing 2 ml of sterile phosphate-buffered solution (10 mmol/L, pH 7.4; Ambion, Thermo Fisher Scientific, Madison, WI, United States) using a pair of flame-sterilized entomological forceps and with the aid of a stereomicroscope (Leica MZ16, 1.6X). The extracted guts were placed in 1.5-ml plastic Eppendorf^®^ tubes containing a DNA shield and stored at –70°C for DNA extraction and sequencing.

### Microbiome Analysis

#### Extraction of DNA

The samples used in this study were analyzed using the ZymoBIOMICS^®^ service performed by Zymo Research (Irvine, CA, United States). DNA was extracted from samples collected and processed with the ZymoBIOMICS™ Service - Targeted Metagenomic Sequencing (Zymo Research, Irvine, CA, United States). The ZymoBIOMICS^®^-96 MagBead DNA Kit (Zymo Research, Irvine, CA, United States) was used to extract DNA.

#### Targeted Library Preparation

Bacterial 16S rRNA gene-targeted sequencing was performed using the Quick-16S™ NGS Library Prep Kit (Zymo Research, Irvine, CA, United States). Bacterial 16S primers amplified the V1-V2 or V3-V4 region of the 16S rRNA gene. These primers have been custom designed by Zymo Research to provide the best coverage of the 16S gene while maintaining high sensitivity. The sequencing library was prepared using a library preparation process in which PCR was performed in real-time PCR instruments to control cycles and prevent/limit PCR chimera formation. The final PCR products were quantified with qPCR fluorescence readings and pooled together based on equal molarity. The final pooled library was cleaned up with the Select-a-Size DNA Clean & Concentrator™ (Zymo Research, Irvine, CA, United States) and then quantified with TapeStation^®^ and Qubit^®^. The final library was sequenced on Illumina^®^ MiSeq™ with a v3 reagent kit (600 cycles). The sequencing was performed with > 10% PhiX spike-in.

### Bioinformatics Analysis

Raw reads were quality-filtered to remove low-quality data and chimeric sequences using Dada2 pipeline (Callahan et al., 2016). The resulting data were analyzed using the Quantitative Insights Into Microbial Ecology (Qiime v.1.9.1) pipeline. Reads were clustered into operational taxonomic units (OTUs) with representative sequences and calculated read counts (abundances) into OTUs at 97% (species-level) sequence identity to compare OTUs abundance between treatments. If an OTU contained fewer than 5 reads, they were omitted from downstream analyzes. Taxonomy assignment was performed using Uclust from Qiime v.1.9. with Greengenes database as reference with a 0.80 confidence threshold.^1^ If applicable, a taxonomy that showed significant abundance between different groups was identified by LEfSe (Segata et al., 2011) using default settings. Alpha-diversity (Shannon diversity) and beta-diversity (Chao1) analyzes were performed with Qiime v.1.9.1 pipeline (Caporaso et al., 2010). We performed a principal coordinates analysis (PCoA) on Bray–Curtis distances using Qiime v.1.9 to compare the microbial community differences between different treatments. Permutational multivariate analysis of variance (PERMANOVA) was applied to Bray-Curtis similarity matrices to compute similarities between groups using PAST statistical software version 2.17. Other analyzes such as heatmaps and abundance plots were performed with internal scripts (see Supplementary 3 and 4).

### Statistical Analysis

Kaplan–Meier survival and Mantel–Haenszel analysis tests were conducted using IBM SPSS Statistical Software version 20.0 to determine the statistically significant differences in the survival of mosquitoes treated with antibiotics and control after treatment with the insecticide. Differences were significant when *p* ≤ 0.05.

## Results

### Mosquito Susceptibility to Pyrethroid Insecticides After Treatment With Antibiotics

**FIGURE 1:**
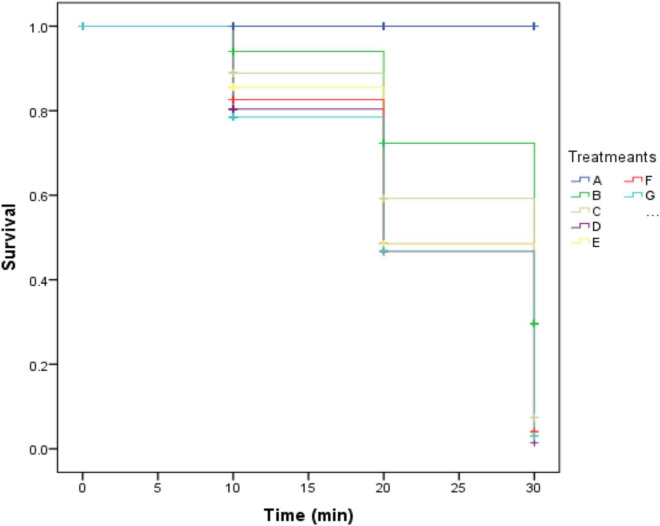
Kaplan-Meier survival curve after exposure to the pyrethroid insecticides permethrin and deltamethrin in an adult population of *Aedes aegypti* using the CDC bottle bioassay (*p* ≤ 0.05). A: fed with sucrose 10%, B: exposed to permethrin. (control group), C: treated with penicillin/streptomycin permethrin exposed, D: treated with gentamicin permethrin exposed, E: exposed to deltamethrin (control group), F: treated with penicillin/streptomycin deltamethrin exposed, and G: treated with gentamicin deltamethrin exposed.

Here, we examined the impact on the mosquito microbiota after treatment with one concentration of two antibiotics including penicillin/streptomycin and gentamicin as described above. As expected, a decrease in the survival rate in mosquitoes exposed to permethrin (30.8%) and deltamethrin (55.8%) was observed compared to mosquitoes that were fed with 10% sucrose and no exposed to insecticides. In turn, a significant difference was observed (*p* ≤ 0.05) between the different groups exposed to permethrin after treatment with penicillin/streptomycin (16%) or gentamicin (28%) compared to control group fed with 10% sucrose exposed to permethrin. Further, no difference was seen in the group of mosquitoes exposed to deltamethrin that were treated with penicillin/streptomycin (2%). Likewise, the group treated with gentamicin showed a decrease in survival (4%) (Figure. 1).

### *Aedes aegypti* Microbial Communities

**FIGURE 2:**
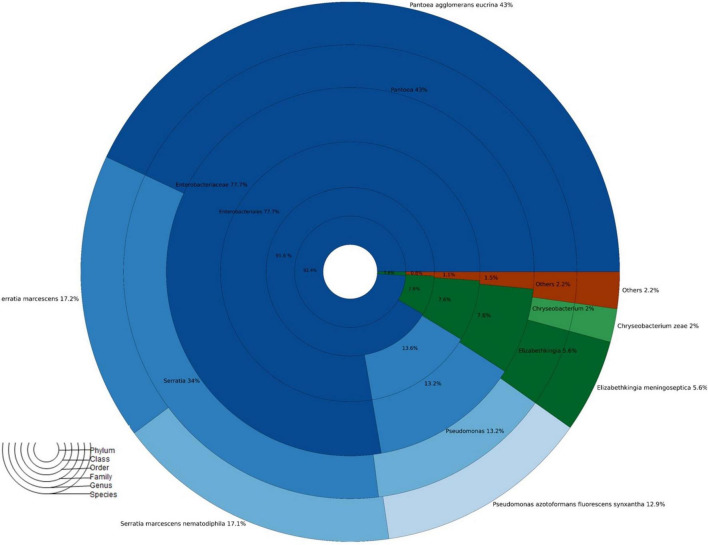
Taxonomic abundance of *A. aegypti* mosquitoes fed 10% sucrose no exposed to insecticides.

A total of 1,48,300 rawseqs were generated from adult *A. aegypti*. Proteobacteria (92.4%) and Bacteroidetes (7.6%) were the most abundant bacterial phyla found in adult mosquito populations fed with 10% sucrose. Briefly, the bacterial diversity was predominantly composed of the classes Gammaproteobacteria (91.6%), Flavobacteria (7.6%), Betaproteobacteria (0.5%), and Alphaproteobacteria (0.3%). Additionally, Enterobacteriales (77.7%), Pseudomonales (13.6%), Flavobacteriales (7.6%), Burkholderiales (0.5%), and Aeromonadales (0.2%) were found as the main bacterial orders. Overall, Enterobacteriaceae (74.7%), Pseudomonadaceae (13.1%), and Flavobacteriaceae (7.6%) were the most abundant families. The genus *Pantoea* (43.0%) was the most abundant, whereas *P. agglomerans* was the most predominant species. Other species found were: *Serratia marcescens* (17.2%), *Serratia marcescens-nemathodiphila* (17.1%), *Pseudomonas azotoformans-fluorescens-synxantha* (12.9%), *Elizabethkingia meningoseptica* (5.6%) and *Chryseobacterium zeae* (2%) (Figure 2).

### Microbiota *Aedes aegypti* Population After Treatment With Antibiotic

Here, the microbiota of *A. aegypti*, when the groups were treated with penicillin/streptomycin, was composed only of the phylum Proteobacteria (99.99%), while in the groups treated with gentamicin was represented by Proteobacteria (96.23%) and Firmicutes (3.77%). Also, the family that was found in gentamicin treatments was *Alcaligenacea* (95.2%), and the main genera were *Bordetella* (95.2%), *Staphylococcus* (3.7%), and *Serratia* (1.1%). In the group treated with penicillin/streptomycin, the main families were *Comammonodaceae* (86%) and *Pseudomonace*ae (13.2%). The genera *Delfia* (86.0%) and *Pseudomonas* (13.36%) were the most abundant in this group (Supplementary Table 1).

### Changes in the Gut Microbiota Composition of *Aedes aegypti* Exposed to Insecticides

In PERMANOVA, pairwise comparisons indicated that the microbiota profiles of mosquitoes treated with antibiotics and later exposed to permethrin were statistically different from those exposed to deltamethrin (*p* ≤ 0.05).

### Exposure to Permethrin

**FIGURE 3:**
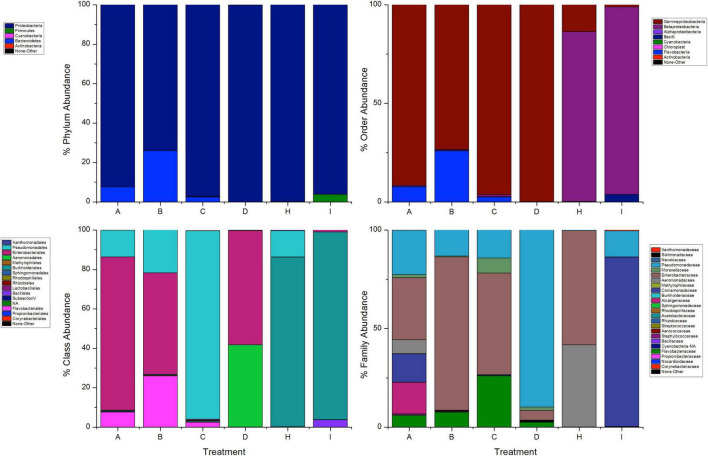
Taxonomic variation between groups of *A. aegypti* exposed to permethrin. A: fed with sucrose 10%, B: exposed to permethrin (control group), C: treated with penicillin/streptomycin permethrin exposed, D: treated with gentamicin permethrin exposed, H: treated with penicillin/streptomycin (not exposed), I: treated with gentamicin (not exposed).

The most abundant phyla in mosquitoes without modification of microbiota that were exposed to permethrin were Proteobacteria (74.1%) and Bacteroidetes (26%). In contrast, Proteobacteria (99.9%) was the dominant phylum in the mosquitoes treated with gentamicin that were exposed to permethrin. In the phylum Proteobacteria, the most representative class in all treatments was Gammaproteobacteria. Families found in mosquitoes that were only exposed to permethrin were Enterobacteriaceae (51.6%), Flavobacteriaceae (25.8%), Pseudomonadaceae (14.20%), and Moraxellaceae (7.6%). For the mosquitoes treated with penicillin/streptomycin and exposed to this insecticide, an increase in the family Pseudomonadaceae (93.9%) and decrease in the family Moraxellaceae (2.4%) was observed. Meanwhile, in mosquitoes treated with gentamicin and exposed to this insecticide, two families predominated Enterobacteriaceae (57.9%) and Aeromonadaceae (41.7%) (Figure 3).

**FIGURE 4:**
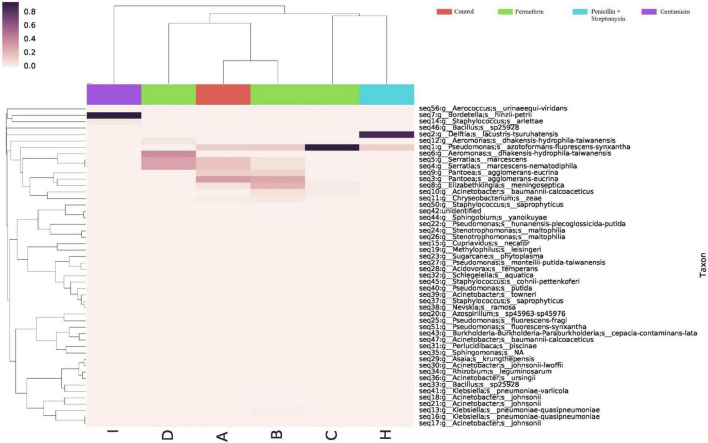
Abundance heatmap of taxa with simple clustering (species) of bacterial communities of *A. aegypti* mosquitoes with permethrin exposure after microbiome suppression. A: fed with sucrose 10%, B: exposed to permethrin (control group), C: treated with penicillin/streptomycin permethrin exposed, D: treated with gentamicin permethrin exposed, H: treated with penicillin/streptomycin (not exposed), I: treated with gentamicin (not exposed).

**FIGURE 5:**
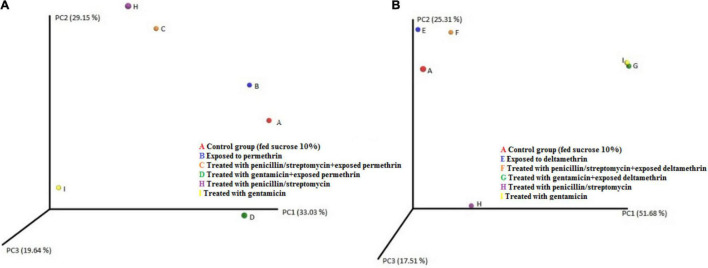
PCoA plot using Bray–Curtis distance showing the distribution of bacterial community composition in *A. aegypti.* **(A)** permethrin exposure **(B)** deltamethrin exposure.

Differences were found at the species level within the groups evaluated. In mosquitoes without modification of microbiota and exposed to permethrin, the most abundant species were *P. agglomerans* (38.4%), followed by *E. meningoseptica* (22.6%) (Figure 4). Diversity profiles showed that only bacterial communities exposed to permethrin were significant (α ≤ 1), according to their non-parametric estimator Chao1 value (21.52; 95% CI = 8.97–23.1), compared to the control group, which showed an estimator Chao1 value (18.70; 95% CI = 6.4–20). This indicated that exposure to the insecticide depended directly on the abundance of the bacterial species. PCoA plot with Bray-Curtis distance comparison showed a difference between the bacterial communities of the groups (*F* = 6.38, *p* = 0.009) with microbiota modification exposed to insecticide including groups treated with penicillin/streptomycin without exposition and groups only exposed to the insecticide (Figure 5). Even though the treatments with penicillin/streptomycin exposed to this insecticide were dominated by only one species, i.e., *P. azotoformans-fluorescens-synxantha* (93.7%), the estimator Chao1 was greater (30.1 95% CI = 1.4–34.1) when compared to the other treatments, so that the species found in greater proportion were *E. meningoseptica* (2.4%) and *A. baumannii-calcoaceticus* (6.1%), and about 16 species showed an abundance of < 1%. On the other hand, there was less diversity (α > 1) in the mosquitoes exposed to insecticide-treated with gentamicin because of the predominance of three species: *Aeromonas dhakensis-hydrophila-taiwanensis* (41.7%), *Serratia marcescens* (28.8%) and *S. marcescens-nematodiphila* (29.1%).

### Exposure to Deltamethrin

**FIGURE 6:**
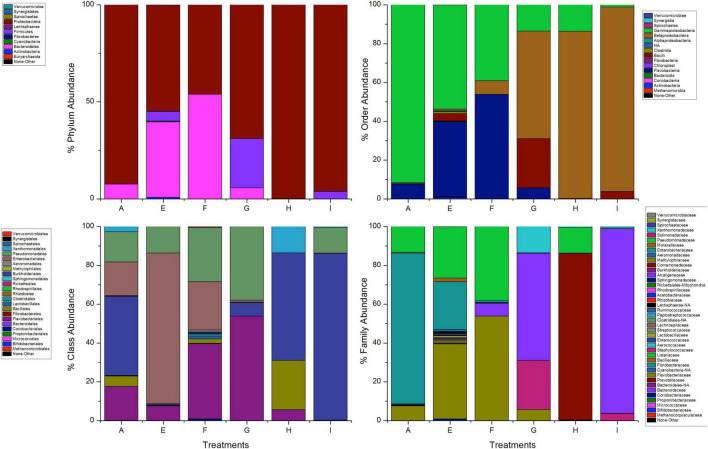
Taxonomic variation between groups of *A. aegypti* exposed to deltamethrin. A: fed with sucrose 10% E: exposed to deltamethrin (control group), F: treated with penicillin/streptomycin deltamethrin exposed, G: treated with gentamicin deltamethrin exposed, H: treated with penicillin/streptomycin (not exposed), I: treated with gentamicin (not exposed).

**FIGURE 7:**
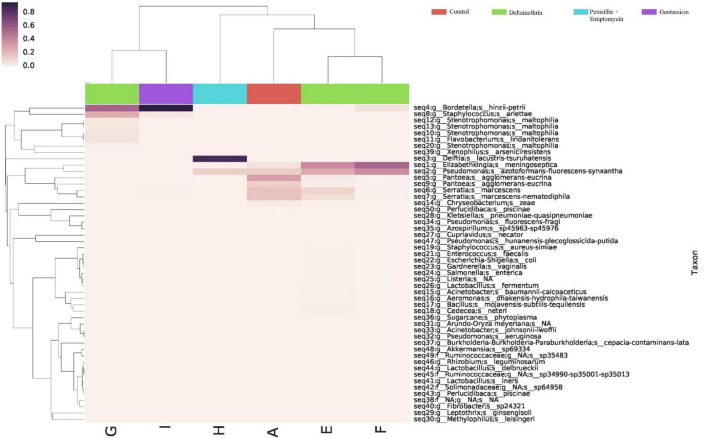
Abundance heatmap of taxa with simple clustering (species) of bacterial communities of *A. aegypti* mosquitoes in deltamethrin exposure after microbiome suppression. A: fed with sucrose 10%, E: exposed to deltamethrin (control group), F: treated with penicillin/streptomycin deltamethrin exposed, G: treated with gentamicin deltamethrin exposed, H: treated with penicillin/streptomycin (not exposed), I: treated with gentamicin (not exposed).

The bacterial communities in the mosquitoes exposed only to deltamethrin were dominated by the phyla Proteobacteria (54.8%) and Bacteroidetes (38.9%). In the same way, the mosquitoes treated with penicillin–streptomycin that were exposed to deltamethrin were represented by Proteobacteria (46.2%) and Bacteroidetes (53.8%). In groups treated with gentamicin and exposed to deltamethrin, the main phyla were Bacteroidetes (5.7%) and Firmicutes (25.3%) (Figure 6). *E. meningoseptica* was found in mosquitoes exposed to deltamethrin and the groups treated with penicillin-streptomycin exposed to this insecticide. The family Staphylococcaceae was only found in the mosquitoes with microbiota modified with penicillin-streptomycin, showing *Staphylococcus arlettae* (25.3%) as the most representative species. Species such as *P. agglomerans-eucrina, S. marcescens*, and *S. marcescens-nematodiphila* complex found in the control group as predominant in this group were found in a lower proportion (Figure 7). In the family Pseudomonadaceae, there was an increase in concentration after treatment with deltamethrin and exposure to penicillin-streptomycin. There was a significant difference in the diversity profiles (α ≤ 1) according to the non-parametric estimator Chao1, which was 74.10 (95% CI = 7.3–82.45), compared to the control group [Chao1 estimator: 18.70 (95% CI = 6.4–20)]. This indicated a substantial increase in the abundance of mosquitoes exposed to deltamethrin. While groups whose microbiota were modified showed less diversity (α > 1) compared to the control group according to Chao1 estimator of 12.63 (95% CI = 1.4–34.1, penicillin-streptomycin) and 10.36 (95% CI = 4.5–11, gentamicin). PCoA plot with Bray–Curtis distance comparison showed differences between bacterial communities of the control group and group of mosquitoes fed with sucrose 10%.

## Discussion

Insects are a broad group of organisms with a large variety of lifestyles, which depends directly on microorganism associations (Douglas, 2014; Guégan et al., 2018). Adaptations of mosquitoes to selective pressure have made it necessary to determine new ways to approach their control. There are only a few studies regarding the effect of insecticides on mosquito microbiota, and thus in this study, we determined the microbiota of a field population of *A. aegypti* in southern Mexico with sequencing techniques and the characterization of key bacteria in response to two pyrethroid insecticides. Previous studies have been conducted to determine the microbiota of mosquitoes in different geographical regions, which have allowed the determination of the microbial diversity of these species of mosquitoes (Ramirez et al., 2012). In our study, the natural microbiota of the *A. aegypti* population (control) was mainly dominated by the phyla Proteobacteria and Bacteriodetes; these results are like those previously reported by Wang et al. (2011). Bacterial populations differ because of the habitat in which they interact as it depends on food, through plants for sugar sources, or through blood in female mosquitoes (Guégan et al., 2018). There are several studies about the microbiota in *A. aegypti* populations of endemic areas of Panama where the predominant phyla are Proteobacteria and Firmicutes (Ramirez et al., 2012).

The predominant species in our control group were *P. agglomerans-eucrina, S. marcescens-nematodiphila, P. azotoformans-fluorescens-synxantha*, and *E. meningoseptica.* Previously, these bacterial genera have been isolated from other species of mosquitoes such as *Anopheles gambiae* and *A. funestus* from Kenia and Mali (Straif et al., 1998). Similarly, in *A. aegypti* (Rockefeller), the genera *Bacillus, Elizabethkingia, Enterococcus, Klebsiella, Pantoea, Serratia*, and *Sphingomonas* (Gusmão et al., 2010; Terenius et al., 2012) have been isolated. In Brazil, the most abundant genera detected in *A. aegypti* populations have been *Pseudomonas, Acinetobacter*, and *Aeromonas* (David et al., 2016). In addition, in *Anopheles* species from Vietnam, *Acinetobacter spp.* has been found to be the main component of the gut microbiota (Ngo et al., 2016). In the same way, microbiota in *Anopheles* has included *Pseudomonas, Comamonas, Acinetobacter, Rhizobium, Burkholderia*, and members of the family Enterobacteriaceae (Tchioffo et al., 2016). The midgut microbiota of *Culex quinquefasciatus* has been shown to harbor bacterial species such as *Acinetobacter junii, Ac. calcoaceticus, Aeromonas culicicola, Bacillus thuringiensis, Microbacterium oxydans, P. agglomerans, P. aeruginosa, Staphylococcus epidermidis*, and *Stenotrophomonas maltophila* (Pidiyar et al., 2004).

Adult mosquitoes may contain a bacterial diversity concentrated mainly in aerobic and anaerobic gram-negative bacteria (Coon et al., 2014, 2016). Many of the related associations between bacterial communities of an insect are known to be mutualists where the host provides bacteria with nutrients and habitat, having selectivity due to the physicochemical conditions present in the host intestine (e.g., alkaline pH, redox potential, oxygen level below 5%, etc.) (Dada et al., 2019). It has been reported that many gram-negative bacteria are frequently found in the middle intestine of vector insects, influencing differential growth by contributing to the modulation of vector competition (Azambuja et al., 2005). In mosquitoes, the bacteria of the phylum Proteobacteria, especially the family Enterobacteriaceae, which are the main components of the middle gut microbiome, can tolerate the redox stress of blood digestion (Jupatanakul et al., 2014). In our study, the most representative species in *A. aegypti* population (control group) was *P. agglomerans*. It is common to find this bacterium as a symbiont in mosquitoes since it is a natural inhabitant of the environment (Segado Arenas et al., 2012; Mayer et al., 2017). In mosquitoes, this bacterium participates in nitrogen fixation, creating a nitrogen-rich environment ideal for the development of eggs and larvae (MacCollom et al., 2009).

The microbiota of our population under study was modified by reducing important enterobacteria species to observe their impact on insecticide response. The populations of *A. aegypti* treated with penicillin/streptomycin and then exposed to permethrin showed a decrease in survival of 16% compared to the mosquitoes not treated. The populations modified with gentamicin decreased by 28% compared to those mosquitoes not treated but exposed to permethrin. The results showed that >90% of bacteria in these treatments were represented by the species *P. azotoformans-fluorescens-synxantha* with penicillin/streptomycin treatment, while in treatments with gentamicin, *A. dhakensis-hydrophila-taiwanensis* and *S. marcescens-nematodiphila* accounted for 29.10%. The strains of *Pseudomonas* are gram-negative, ubiquitous bacteria, characterized by primary nutritional needs and presence in various environments (soil, decaying organic material, atmospheric dust, vegetation, and water), with a wide range of plants and animals (Andreani et al., 2015). This genus has been found as part of the bacterial community in insects. Some research points to the toxic effectiveness of *P. aeruginosa* in the larvae of different mosquito species of the genera *Aedes, Anopheles, Culex*, and *Culiseta. In vivo* experiments with *P. fluoresences* cultures have found effectiveness against pupae of three mosquito species: *An. stephensi, Cx. quinquefasciatus* and *Ae. aegypti* (Prabakaran et al., 2009). Similarly, Nabar and Lokegaonkar (2015) reported the larvicidal power of metabolites extracted from *Pseudomonas* spp. The species *P. fluorescens* has active metabolites against the larvae and pupae of *Cx quinquefasciatus* mosquitoes (Sadanandane et al., 2003; Brammacharry and Paily, 2012).

On the other hand, with the modification of the microbiota with gentamicin, we found the complex *A. dhakensis-hydrophila-taiwanensis.* This genus of gram-negative bacteria inhabits many environments (aquatic, fish, food, domesticated pets, invertebrate species, birds, ticks and insects, and natural soils) (Figueras et al., 2005; Janda and Abbott, 2010), and has been isolated from the intestines of *Cx. quinquefasciatus* (Pidiyar et al., 2002, 2004). Similarly, the species *A. hydrophila* has been reported to possess chitinolytic enzymes with activity against *Cx. quinquefasciatus* under laboratory conditions (Halder et al., 2012). As we observed in our results, when modifying the microbiota, enterobacteria were eliminated, indicating that these bacteria may play an important role in maintaining the response to insecticides. Certain bacterial communities such as *Pseudomonas* spp. and *Aeromonas spp*. were increased; it has been reported that some of these bacteria may produce metabolites that can compromise the physiological functioning of the mosquitoes when they are exposed to insecticides. Therefore, we could associate this type of response to this event resulting in decreased survival in our results.

The response with deltamethrin was different from what we observed in the permethrin group. There was a non-significant 2% decrease in mosquito survival with exposure to deltamethrin and penicillin/streptomycin treatment and a reduction of 4% with gentamicin compared to those whose microbiota was not modified. However, we could observe a change in the microbiota populations exposed to deltamethrin. The mosquitoes treated with penicillin/streptomycin exposed to this insecticide showed as predominant species *E. meningoseptica* and *P. azotoformans-fluorescens-synxantha*, while *B. hinzii-petrii* and *S. arletta* in those exposed to gentamicin. *E. meningoseptica* is a non-motile gram-negative bacillus, ubiquitous in nature (Kim et al., 2005). The members of the genus *Elizabethkingia* are found in wet habitats, particularly in water supplies (Kämpfer et al., 2011). In mosquitoes, it has been reported that this bacterium can modulate anti*-Plasmodium* effects, thus prolonging the lifespan of the infected mosquito vector (Akhouayri et al., 2013). In addition, it has been consistently found in laboratory conditions to be an important part of the functionality of mosquitoes (Wang et al., 2011). *E. anophelisis* is known to be a dominant bacterium in the gut microbiota in the mosquito *A. gambiae* (Rani et al., 2009; Gusmão et al., 2010; Kämpfer et al., 2011; Boissière et al., 2012; Akhouayri et al., 2013), and *E. meningoseptica* has been found in *Cx. quinquefasciatus* (Terenius et al., 2012). Similarly, the genus *Elizabethkingia* was detected in 68% of mosquito populations collected in Cameroon (Chen et al., 2015). The species *Bordetella sp.*, which was the most abundant bacterium in the modification with gentamicin, is found in various aquatic environments and terrestrial environmental sources, also associated with plants (Shamim et al., 2019). The genus *Bordetella* has been isolated from adult mosquitoes of *An. stephensi* (Chavshin et al., 2012). Although these species are not common among microbes in mosquitoes, they can be found in small amounts in the intestinal ecosystem or tissues of mosquitoes, having an ecological connection to behavior and protection against adverse conditions (Kukutla et al., 2013).

Recent studies have demonstrated the role of symbiotic bacteria in detoxification processes (Itoh et al., 2018). This was observed in arthropods with phenothion applied in agricultural fields which were shown to be degraded by *Pseudomonas, Flavobacterium*, and *Burkholderia* (Kikuchi et al., 2012). *Bacillus cereus*, isolated from the guts of the *P. xylostella* moth, has been found to have a high degradation capability for the pesticide indoxacarb (Ramya et al., 2016a). Similarly, *Enterobacter asburiae* and *P. agglomerans* were found to degrade acephalate (Ramya et al., 2016b). Also, *Citrobacter* sp. was isolated from the intestine of *B. dorsalis*, capable of hydrolyzing trichlorphon (McFall-Ngai et al., 2013). Recently, a study determined the effects of pyrethroid insecticides in *An. albimanus* microbiota, finding differences with respect to mosquitoes not exposed to pyrethroids, determining *Klebsiella, Pantoea*, and *Asia* as key species in resistance to pyrethroids (Dada et al., 2019). In our study, we did not find species with evidence of metabolism of deltamethrin; however, we observed that with exposure to deltamethrin, there were different patterns of bacterial profiles compared to those seen with exposure to permethrin, which could influence the detoxification process. Our results clearly showed a different response to two insecticides from the same family, previously noted in Dada et al. (2019)’s study, where they obtained different microbiota patterns against pyrethroid insecticides. It is very important in future investigations to study the participation of key bacteria in the mechanisms of detoxification in mosquitoes since there is limited information.

## Conclusion

The results of this study describe the microbiota of *A. aegypti*, which were dominated by species of *Proteobacteria* and *Bacteroidetes.* In nature, insects frequently face unfavorable environmental conditions that can alter symbiotic bacteria. In this study, we observed that changes in the microbiota affect the mosquito’s response to exposure to pyrethroid insecticides. Symbiont microorganisms associated with mosquitoes have been shown to play a key role in response to insecticide exposure. The search for key symbionts related to response to insecticides will allow us to understand mosquito physiology and then create tools and new targets for controlling mosquito populations. The development of this research will generate basic knowledge of key bacteria of each population analyzed.

## Data Availability Statement

The datasets presented in this study can be found in online repositories. The names of the repository/repositories and accession number(s) can be found below: National Center for Biotechnology Information (NCBI) BioProject database under accession numbers OK646404-OK646460 entitled “Uncultured Prokaryotic 16S rRNA/Microbiome Aedes aegypti Group 1” and OK648490-OK648570 entitled “Uncultured Prokaryotic 16SrRNA/Group 2”.

## Ethics Statement

Ethical review and approval were not required for the animal study because the manuscript presents research results on invertebrate animals (*Aedes aegypti*).

## Author Contributions

All authors contributed to the Replication of experiments, data analysis, and manuscript review.

## Conflict of Interest

The authors declare that the research was conducted in the absence of any commercial or financial relationships that could be construed as a potential conflict of interest.

## Publisher’s Note

All claims expressed in this article are solely those of the authors and do not necessarily represent those of their affiliated organizations, or those of the publisher, the editors and the reviewers. Any product that may be evaluated in this article, or claim that may be made by its manufacturer, is not guaranteed or endorsed by the publisher.

## Footnotes

1
https://greengenes.secondgenome.com/
