# Supplementary material for: Suppression of Midgut Microbiota Impact Pyrethroid Susceptibility in Aedes aegypti
Source: Front Microbiol. 2022 Aug 1;13:761459. doi: 10.3389/fmicb.2022.761459 (PMC9376455; doi:10.3389/fmicb.2022.761459)
Supplement: Supplementary file 1 [file Table_1.DOCX]

**TABLE 1.** Bacterial found after treatment with antibiotics compared with no antibiotic mosquitoes in *Ae. aegypti.*

| no antibiotic | penicillin/streptomycin | gentamicin | Treatments |
| --- | --- | --- | --- |
| **FAMILY** | | |  |
| 7.6 |  |  | Flavobacteriaceae |
|  |  | 3.7 | Staphylococcaceae |
|  | 86 |  | Cormmaminadaceae |
| 0.2 |  |  | Aeromonadaceae |
| 78 |  | 1 | Enterobacteriaceae |
| 0.5 |  |  | Moraxellaceae |
| 13.6 | 13.2 |  | Pseudomonadaceae |
|  | 0.2 | 95.2 | Alcaligenaceae |
| 0.1 | 0.6 | 0.1 | Others (<1%) |
| **GENUS** | | |  |
| 2 |  |  | *Chryseobacterium* |
| 5.6 |  |  | *Elizabethkingia* |
|  |  | 3.7 | *Staphylococcus* |
| 0.4 |  | 95.2 | *Bordetella* |
|  | 86 |  | *Delftia* |
| 43 |  |  | *Pantoea* |
| 34.4 |  | 1.1 | *Serratia* |
| 13.2 | 13.2 |  | *Pseudomonas* |
| 1.4 | 0.8 |  | *Others (<1%)* |
| **SPECIES** | | |  |
| 2 |  |  | *Chryseobacterium zeae* |
| 5.6 |  |  | *Elizabethkingia meningoseptica* |
|  |  | 3.7 | *Staphylococcus arlettae* |
| 0.4 |  | 95.2 | *Bordetella hinzii-petrii* |
|  | 86 |  | *Delftia lacustris-tsuruhatensis* |
| 43 |  |  | *Pantoea agglomerans-eucrina* |
| 17.2 |  | 0.6 | *Serratia marcescens* |
| 17.1 |  | 0.5 | *Serratia marcescens-nematodiphila* |
| 12.9 | 13.1 |  | *Pseudomonas azotoformans-fluorescens-synxantha* |
| 1.8 | 0.9 |  | *Others (<1%)* |
