# Supplementary material for: Suppression of Midgut Microbiota Impact Pyrethroid Susceptibility in Aedes aegypti
Source: Front Microbiol. 2022 Aug 1;13:761459. doi: 10.3389/fmicb.2022.761459 (PMC9376455; doi:10.3389/fmicb.2022.761459)
Supplement: Supplementary file 4 [file Data_Sheet_3.DOCX]

a.header:link { color: black; font-family:Arial,Verdana; font-size:11; font-weight: bold;}

a.header:visited { color: black; font-family:Arial,Verdana; font-size:11; font-weight:bold;}

a.header:hover { color: black; font-family:Arial,Verdana; font-size:11; font-weight:bold;}

a.header:active { color: black; font-family:Arial,Verdana; font-size:11; font-weight:bold;}

a:link { color: blue;}

a:visited { color: blue;}

a:hover { color: blue;}

a:active { color: blue; }

a.text_tree:link { color: black; font-family:"Courier New", Courier, monospace; font-size:10px; font-weight: bold; text-decoration: none}

a.text_tree:visited { color: black; font-family:"Courier New", Courier, monospace; font-size:10px; font-weight: bold; text-decoration: none}

a.tree_scale:link { color: red; font-family:"Courier New", Courier, monospace; font-size:10px; font-weight: bold; text-decoration: none; background-color: white;}

a.tree_scale:visited { color: red; font-family:"Courier New", Courier, monospace; font-size:10px; font-weight: bold; text-decoration: none; background-color: white;}

a.tree_scale:hover { color: white; font-family:"Courier New", Courier, monospace; font-size:10px; font-weight: bold; text-decoration: none; background-color: red;}

td.header-attn { background-color: yellow; border: 1px solid #DDDDDD; color: black; font-family:Arial, Verdana; font-size:11; font-weight:bold;}

td.header-last { background-color: #fdfdf4; border: 1px solid #DDDDDD; color: black; font-family:Arial, Verdana; font-size:11; font-weight:bold;}

td.header1 { background-color: #e4f7fd; border: 1px solid #DDDDDD; color: black; font-family:Arial, Verdana; font-size:11; font-weight:bold;}

td.header2 { background-color: #e4fdf8; border: 1px solid #cccccc; color: black; font-family:Arial, Verdana; font-size:11; font-weight:bold;}

td.header3 { background-color: #e4fdf5; border: 1px solid #cccccc; color: black; font-family:Arial, Verdana; font-size:11; font-weight:bold;}

td.header4 { background-color: #e4e8fd; border: 1px solid #cccccc; color: black; font-family:Arial, Verdana; font-size:11; font-weight:bold;}

td.header5 { background-color: #f5f7ef; border: 1px solid #DDDDDD; color: black; font-family:Arial, Verdana; font-size:11; font-weight:bold;}

td.header { background-color: #eff4f7; border: 1px solid #DDDDDD; color: black; font-family:Arial, Verdana; font-size:10; font-weight:bold;}

td.normal { text-align:center;border-width:medium;border-style:solid;border: 1px solid #DDDDDD; color: black; font-family:Arial, Verdana; font-size:9; font-weight:bold;}

td.nav_sel { background-color: red; border: 1px solid #DDDDDD; color: white; font-family:Arial, Verdana; font-size:11; font-weight:bold;}

td.nav_grey { background-color: eeeeee; border: 1px solid #DDDDDD; color: red; font-family:Arial, Verdana; font-size:11; font-weight:bold;}

td.nav_other { background-color: white; border: 1px solid #DDDDDD; color: red; font-family:Arial, Verdana; font-size:11; font-weight:bold;}

td.nav_tax_sel { background-color: red; border: 1px solid #DDDDDD; color: white; font-family:Arial, Verdana; font-size:10; font-weight:bold; font-style: italic;}

td.nav_tax_grey { background-color: eeeeee; border: 1px solid #DDDDDD; color: red; font-family:Arial, Verdana; font-size:10; font-weight:bold; font-style: italic;}

td.nav_tax_other { background-color: white; border: 1px solid #DDDDDD; color: red; font-family:Arial, Verdana; font-size:10; font-weight:bold; font-style: italic;}

a.nav:link { color: red; font-family:Arial,Verdana; font-size:11; font-weight: bold;}

a.nav:visited { color: red; font-family:Arial,Verdana; font-size:11; font-weight:bold;}

a.nav:hover { color: red; font-family:Arial,Verdana; font-size:11; font-weight:bold;}

a.nav:active { color: red; font-family:Arial,Verdana; font-size:11; font-weight:bold;}

a.nav_tax:link { color: black; font-family:Arial,Verdana; font-size:10; font-weight: bold; font-style: italic;}

a.nav_tax:visited { color: black; font-family:Arial,Verdana; font-size:10; font-weight:bold; font-style: italic;}

a.nav_tax:hover { color: black; font-family:Arial,Verdana; font-size:10; font-weight:bold; font-style: italic;}

a.nav_tax:active { color: black; font-family:Arial,Verdana; font-size:10; font-weight:bold; font-style: italic;}

td.smheader-last { background-color: #fdfdf4; border: 1px solid #DDDDDD; color: black; font-family:Arial, Verdana; font-size:7; font-weight:bold;}

td.smheader { background-color: #eff4f7; border: 1px solid #DDDDDD; color: black; font-family:Arial, Verdana; font-size:7; font-weight:bold;}

td.smbheader-last { background-color: #fdfdf4; border: 1px solid #DDDDDD; color: black; font-family:Arial, Verdana; font-size:9; font-weight:bold;}

td.smbheader { background-color: #eff4f7; border: 1px solid #DDDDDD; color: black; font-family:Arial, Verdana; font-size:9; font-weight:bold;}

td.smcolor { background-color: #fcfde6; border: 0px solid #DDDDDD; color: black; font-family:Arial,Verdana; font-size:7; text-align: center; }

.smnorm { color: black; font-family:Arial,Verdana; font-size:6;}

.bsmnorm { color: black; font-family:Arial,Verdana; font-size:6; background-color: #00ffff}

.csmnorm { color: black; font-family:Arial,Verdana; font-size:6; text-align: center; }

.cbsmnorm { color: black; font-family:Arial,Verdana; font-size:6; background-color: #00ffff; text-align: center;}

.smh_norm { color: black; font-family:Arial,Verdana; font-size:7;}

.smhotu_norm { color: black; font-family:Arial,Verdana; font-size:6;}

.smv_header-last { background-color: #fdfdf4; border: 1px solid #DDDDDD; color: black; font-family:Arial, Verdana; font-size:9; font-weight:bold;}

.smv_header { background-color: #eff4f7; border: 1px solid #DDDDDD; color: black; font-family:Arial, Verdana; font-size:9; font-weight:bold;}

.smv_norm { color: black; font-family:Arial,Verdana; font-size:9; text-align: center;}

.smvn_norm { color: black; font-family:Arial,Verdana; font-size:9; }

td.sm_lm_header-last { background-color: #fdfdf4; border: 1px solid #DDDDDD; color: black; font-family:Arial, Verdana; font-size:8; font-weight:bold;}

td.sm_lm_header { background-color: #eff4f7; border: 1px solid #DDDDDD; color: black; font-family:Arial, Verdana; font-size:8; font-weight:bold;}

.sm_lm_norm { color: black; font-family:Arial,Verdana; font-size:6;}

.sm_lmh_norm { color: black; font-family:Arial,Verdana; font-size:9;}

.smcnorm { color: black; font-family:Arial,Verdana; font-size:7; text-align: center;}

.treeticks { font-family: "Courier New", Courier, monospace; font-size: 8; font-weight: bold;}

.tree { color: black; font-family: "Courier New", Courier, monospace; font-size: 8px; }

td.solidheader { background-color: #FFF200; border: 1px solid #FFF200; color: black; font-family:Arial, Verdana; font-size:11; font-weight:bold;}

.cheader { background-color: #FFFDC9; border: 1px solid #FFF200; color: black; font-family:Arial, Verdana; font-size:11; font-weight:bold;}

td.section {background-color: #dfe0c0; border: 1px solid #999999; color: black; font-family:Arial, Verdana; font-size:14; font-weight:bold;}

.normal { color: black; font-family:Arial,Verdana; font-size:11; font-weight:normal;}

.normal_red { color: red; font-family:Arial,Verdana; font-size:11; font-weight:normal;}

.copy { color: red; font-family:Arial,Verdana; font-size:11; font-weight:bold; font-style: italic;}

.sm_bnorm { color: black; font-family:Arial,Verdana; font-size:10; font-weight:bold;}

.ntitle { color: black; font-family:Arial,Verdana; font-size:11; font-weight:bold;}

.ntotal { color: black; font-family:Arial,Verdana; font-size:11; font-weight:bold; font-style: italic;}

.tiny { background-color: #eeeeee; font-family: "Courier New", Courier, monospace; font-size: 10px; font-weight: bold; text-align: center;}

.tinyh { font-family: "Courier New", Courier, monospace; font-size: 10px; font-weight: bold; text-align: center}

.tinyt { font-family: "Arial"; font-size: 8px; font-weight: bold; text-align: left}

.tinytc { font-family: "Arial"; font-size: 8px; font-weight: bold; text-align: center; width: 20px}

.smh_key_norm { color: black; font-family:Arial,Verdana; font-size:10;}
